# Exploring the molecular basis of insecticide resistance in the dengue vector *Aedes aegypti*: a case study in Martinique Island (French West Indies)

**DOI:** 10.1186/1471-2164-10-494

**Published:** 2009-10-26

**Authors:** Sébastien Marcombe, Rodolphe Poupardin, Frederic Darriet, Stéphane Reynaud, Julien Bonnet, Clare Strode, Cecile Brengues, André Yébakima, Hilary Ranson, Vincent Corbel, Jean-Philippe David

**Affiliations:** 1 Laboratoire de Lutte contre les Insectes Nuisibles (LIN), Institut de Recherche Pour le Développement (IRD) Montpellier, France; 2 Laboratoire d'Ecologie Alpine (LECA, UMR 5553 CNRS - Université de Grenoble), Grenoble, France; 3 Vector Research group, Liverpool School of Tropical Medicine, Liverpool, UK; 4 Centre de démoustication, Conseil général de la Martinique, Fort de France, Martinique, France

## Abstract

**Background:**

The yellow fever mosquito *Aedes aegypti *is a major vector of dengue and hemorrhagic fevers, causing up to 100 million dengue infections every year. As there is still no medicine and efficient vaccine available, vector control largely based on insecticide treatments remains the only method to reduce dengue virus transmission. Unfortunately, vector control programs are facing operational challenges with mosquitoes becoming resistant to commonly used insecticides. Resistance of *Ae. aegypti *to chemical insecticides has been reported worldwide and the underlying molecular mechanisms, including the identification of enzymes involved in insecticide detoxification are not completely understood.

**Results:**

The present paper investigates the molecular basis of insecticide resistance in a population of *Ae. aegypti *collected in Martinique (French West Indies). Bioassays with insecticides on adults and larvae revealed high levels of resistance to organophosphate and pyrethroid insecticides. Molecular screening for common insecticide target-site mutations showed a high frequency (71%) of the sodium channel 'knock down resistance' (*kdr*) mutation. Exposing mosquitoes to detoxification enzymes inhibitors prior to bioassays induced a significant increased susceptibility of mosquitoes to insecticides, revealing the presence of metabolic-based resistance mechanisms. This trend was biochemically confirmed by significant elevated activities of cytochrome P450 monooxygenases, glutathione S-transferases and carboxylesterases at both larval and adult stages. Utilization of the microarray *Aedes Detox Chip *containing probes for all members of detoxification and other insecticide resistance-related enzymes revealed the significant constitutive over-transcription of multiple detoxification genes at both larval and adult stages. The over-transcription of detoxification genes in the resistant strain was confirmed by using real-time quantitative RT-PCR.

**Conclusion:**

These results suggest that the high level of insecticide resistance found in *Ae. aegypti *mosquitoes from Martinique island is the consequence of both target-site and metabolic based resistance mechanisms. Insecticide resistance levels and associated mechanisms are discussed in relation with the environmental context of Martinique Island. These finding have important implications for dengue vector control in Martinique and emphasizes the need to develop new tools and strategies for maintaining an effective control of *Aedes *mosquito populations worldwide.

## Background

Every year, 50 to 100 million dengue infections world-wide causing from 20,000 to 25,000 deaths from dengue and hemorrhagic fever are recorded [1]. As there is still no medicine and efficient vaccine available, vector control by the recourse of environmental management, educational programs and the use of chemical and biological agents, remains the only method to reduce the risk of dengue virus transmission [1]. Unfortunately, most of dengue vector control programs implemented worldwide are facing operational challenges with the emergence and development of insecticide resistance in *Ae. aegypti *[2] and *Ae. albopictus *[3]. Resistance of *Ae. aegypti *to insecticides has been reported in many regions including South east Asia [4,5], Latin America [6] and the Caribbean [7].

Inherited resistance to chemical insecticides in mosquitoes is mainly the consequence of two distinct mechanisms: the alteration of target sites inducing insensitivity to the insecticide (target-site resistance) and/or an increased metabolism of the insecticide (metabolic-based resistance) [8]. Metabolic-based resistance involves the bio-transformation of the insecticide molecule by enzymes and is now considered as a key resistance mechanism of insects to chemical insecticides [8,9]. This mechanism may result from two distinct but additive genetic events: *i*) a mutation of the enzyme protein sequence leading to a better metabolism of the insecticide, and/or *ii*) a mutation in a non-coding regulatory region leading to the over-production of an enzyme capable of metabolizing the insecticide. So far, only the second mechanism has been clearly associated with the resistant phenotype in mosquitoes. Three large enzyme families, the cytochrome P450 monooxygenases (P450s), glutathione S-transferases (GSTs) and carboxy/cholinesterases (CCEs) have been implicated in the metabolism of insecticides [8,10-12]. The rapid expansion and diversification of these so-called 'detoxification enzymes' in insects is likely to be the consequence of their adaptation to a broad range of natural xenobiotics found in their environment such as plant toxins [13]. These enzymes have also been involved in mosquito response to various anthropogenic xenobiotics such as heavy metals, organic pollutants and chemical insecticides [14-16].

Although identifying metabolic resistance is possible by toxicological and biochemical techniques, the large panel of enzymes potentially involved together with their important genetic and functional diversity makes the understanding of the molecular mechanisms and the role of particular genes a challenging task. As more mosquito genomes have been sequenced and annotated [17,18], the genetic diversity of genes encoding mosquito detoxification enzymes has been unravelled and new molecular tools such as the *Aedes *and *Anopheles *'detox chip' microarrays allowing the analysis of the expression pattern of all detoxification genes simultaneously have been developed [19,20]. These specific microarrays were successfully used to identify detoxification genes putatively involved in metabolic resistance in various laboratory and field-collected mosquito populations resistant to insecticides [19-24].

In Latin America and the Caribbean, several *Ae. aegypti *populations show strong resistance to pyrethroid, carbamate and organophosphate insecticides correlated with elevated activities of at least one detoxification enzyme family [25-28]. In addition, several points of non-synonymous mutations in the gene encoding the trans-membrane voltage-gated sodium channel (*kdr *mutations) have been described and showed to confer resistance to pyrethroids and DDT [27,29].

Several questions remain concerning the impact of insecticide resistance on the efficacy of vector control operations. In Martinique (French West Indies), high levels of resistance to the organophosphate temephos and the pyrethroid deltamethrin were reported. This resistance was characterized by an important reduction of both mosquito knock-down and mortality levels after thermal-fogging with deltamethrin and P450-inhibitor synergized pyrethroids, indicating that resistance was negatively impacting on control programmes and that this resistance was conferred, at least in part, by elevated cytochrome P450 activity [30].

In this study, we explored the mechanisms conferring insecticide resistance in an *Ae. aegypti *population from Martinique island. Larval bioassays and adult topical applications were used to determine the current resistance level of this population to insecticides. The presence of metabolic-based resistance mechanisms was investigated by exposing mosquitoes to enzyme inhibitors prior to bioassays with insecticides and by measuring representative enzyme activities of each detoxification enzyme family. At the molecular level, the frequency of the target-site *kdr *mutation was investigated and a microarray approach followed by quantitative real-time RT-PCR validation was used to identify detoxification genes putatively involved in metabolic resistance. Results from this study will help to implement more effective resistance management strategies in this major disease vector in the future.

## Results

Larval bioassays (Table 1) showed that the Vauclin strain is far less affected by temephos than the susceptible Bora-Bora strain (RR_50_of 44-fold and RR_95 _of 175-fold). In the susceptible strain, temephos toxicity was not significantly increased in the presence of detoxification enzyme inhibitors (PBO, DEF and DMC). By contrast, the level of resistance to temephos of the Vauclin strain was significantly reduced in the presence of PBO, DEF and DMC (from 175 to 60, 44 and 109-fold respectively for RR_95_) indicating the involvement of P450s, CCEs and in a lesser extent GSTs in the resistance of larvae to temephos.

**Table 1:** Insecticidal activity of temephos with and without enzyme inhibitors on larvae of *Aedes aegypti *Vauclin and Bora-Bora strains

**Strain**	**Enzyme inhibitor**	**Slope** **(± SE)**	**LC_50 _(μg/L)** **(95% CI)**	**LC_95 _(μg/L)** **(95% CI)**	**RR_50_** **(95% CI)**	**RR_95_** **(95% CI)**	**SR_50_** **(95% CI)**	**SR_95_** **(95% CI)**
	-	8.49 (0.45)	3.7 (3.6-3.8)	5.7 (5.5-6)	-	-	-	-
**Bora-Bora**	PBO	8.28 (0.67)	4.2 (4-4.4)	6.7 (6.4-7)	-	-	0.87 (0.74-1.03)	0.87 (0.74-1.03)
	DEF	8.13 (0.44)	3.3 (3.2-3.4)	5.3 (5.1-5.6)	-	-	1.10 (0.98-1.24)	1.10 (0.98-1.24)
	DMC	11.16 (0.54)	4.3 (4.2-4.4)	6.0 (5.8-6.2)	-	-	**0.86** **(0.79-0.94)**	0.96 (0.81-1.14)
	
	-	2.08	160	1000	**44**	**175**	-	-
		(0.08)	(150-180)	(870-1180)	**(40-48)**	**(150-205)**		
	PBO	3.60	140	400	**33**	**60**	**1.16**	**2.52**
**Vauclin**		(0.24)	(130-150)	(360-450)	**(29-38)**	**(51-71)**	**(1.05-1.29)**	**(2.16-2.95)**
	DEF	3.00	68	240	**21**	**44**	**2.37**	**4.27**
		(0.16)	(64-72)	(210-270)	**(18-22)**	**(38-52)**	**(2.18-2.57)**	**(3.64-5)**
	DMC	2.05	103	650	**24**	**109**	**1.57**	**1.57**
		(0.11)	(92-110)	(560-790)	**(22-27)**	**(92-129)**	**(1.39-1.79)**	**(1.39-1.79)**

Resistant ratios RR_50 _and RR_95 _were obtained by calculating the ratio between the LC_50 _and LC_95 _between Vauclin and Bora-Bora strains; Synergism ratios SR_50 _and SR_95 _were obtained by calculating the ratio between LC_50 _and LC_95 _with and without enzyme inhibitor. (CI): Confidence Interval. Significant RR and SR are shown in bold.

Topical applications of the pyrethroid insecticide deltamethrin on adults of each strain (Table 2) revealed that the Vauclin strain is also highly resistant to deltamethrin (RR_50 _of 56-fold and RR_95 _of 76-fold). In both strains, the toxicity of deltamethrin increased significantly in the presence of detoxification enzyme inhibitors, however only PBO and DMC induced higher synergistic effects in the Vauclin strain than in the susceptible Bora-Bora strain (SR_50 _of 9.94 and 3.76 respectively). In the Vauclin strain, PBO and DMC significantly reduced the resistance level (from 76-fold to 41-fold and 43-fold respectively for RR_95_), indicating a significant role of P450s and GSTs in the resistance of adults to deltamethrin.

**Table 2:** Insecticidal activity of deltamethrin with and without enzyme inhibitors on adults of *Aedes aegypti *Vauclin and Bora-Bora strains

**Strain**	**Enzyme inhibitor**	**Body weight (mg)**	**Slope** **(± SE)**	**LD_50 _(μg/L)** **(95% CI)**	**LD_95 _(μg/L)** **(95% CI)**	**RR_50_** **(95% CI)**	**RR_95_** **(95% CI)**	**SR_50_** **(95% CI)**	**SR_95_** **(95% CI)**
**Bora-Bora**	-	2.12	3.31 (0.27)	18 (16-19)	55 (47-69)	-	-	-	-
	PBO	2.27	3.65 (0.34)	3.4 (3.1-3.7)	9.5 (8.1-12.1)	-	-	**5.2** **(4.52-5.98)**	**5.79** **(4.30-7.81)**
	DEF	2.44	2.41 (0.27)	3.4 (3-3.9)	16 (12-25)	-	-	**5.12** **(4.48-5.86)**	**3.35** **(2.42-4.64)**
	DMC	2.39	2.94 (0.22)	7.3 (6.6-8.1)	27 (22-34)	-	-	**2.41** **(2.11-2.76)**	**2.09** **(1.57-2.78)**
	
**Vauclin**	-	2.65	2.61	990	4210	**56**	**76**	-	-
			(0.19)	(880-1100)	(3470-5380)	**(49-64)**	**(58-99)**		
	PBO	2.27	2.78	99	390	**29**	**41**	**9.94**	**10.89**
			(0.17)	(91-108)	(330-470)	**(26-33)**	**(31-53)**	**(8.79-11.23)**	**(8.64-13.72)**
	DEF	2.25	2.14	170	1000	**49**	**60**	**5.81**	**4.23**
			(0.22)	(150-190)	(750-1510)	**(43-56)**	**(43-86)**	**(5.08-6.65)**	**(3.16-5.66)**
	DMC	2.56	2.57	260	1150	**36**	**43**	**3.76**	**3.68**
			(0.16)	(240-290)	(950-1460)	**(32-40)**	**(33-57)**	**(3.35-4.23)**	**(2.86-4.72)**

Resistant ratios RR_50 _and RR_95 _were obtained by calculating the ratio between the LD_50 _and LD_95 _between Vauclin and Bora-Bora strains; Synergism ratios SR_50 _and SR_95 _were obtained by calculating the ratio between LD_50 _and LD_95 _with and without enzyme inhibitor. (CI): Confidence Interval. Significant RR and SR are shown in bold.

**Figure 1:**
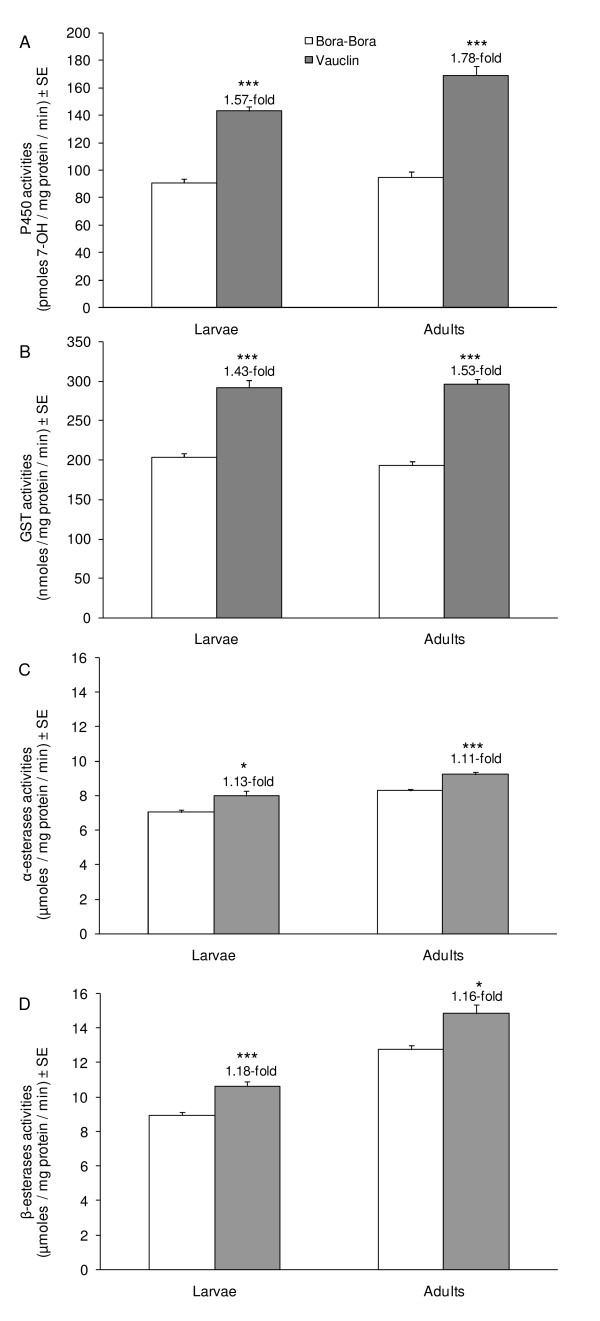
**Comparison of detoxification enzymes activities between the insecticide-resistant strain Vauclin and the susceptible strain Bora-Bora**. A) P450 activities were measured with the ECOD method [63] on 20 μg microsomal proteins after 15 min and expressed as pmol of 7-OH produced/mg microsomal protein/minute (± SE). B) GST activities were measured with the CDNB method [64] on 200 μg cytosolic proteins during 1 min and expressed as nmol of conjugated CDNB/μg protein/min (± SE). α-esterase (C) and β-esterase (D) activities were measured with the naphthyl acetate method [65] on 30 μg cytosolic proteins after 15 min and expressed as μmol α- or β-naphthol produced/mg protein/minute (± SE). For each strain and each life stage, 3 independent biological replicates were analyzed and measures were repeated 15, 15 and 30 times for P450, GST and esterase activities respectively. Statistical comparison of enzyme activities between the Vauclin and Bora-Bora strains were performed at each life stage separately with a Mann and Whitney's test (* p < 0.05, ** p < 0.01, *** p < 0.001).

Comparison of constitutive detoxification enzyme activities between the susceptible strain Bora-Bora and the insecticide-resistant Vauclin strain revealed significant differences at both larval and adult stages (Figure 1). P450 activities were elevated in both larvae and adults of the Vauclin strain (1.57-fold and 1.78-fold respectively with P < 0.001 at both life stages). Similarly, GST activities were found elevated in larvae and adults of the Vauclin strain (1.43-fold and 1.53-fold respectively with P < 0.001 at both life stages). Finally, α- and β-carboxylesterase activities were also found slightly elevated in the Vauclin strain in larvae (1.13-fold and 1.18-fold with P < 0.05 and P < 0.001 respectively) and adults (1.11-fold and 1.16-fold with P < 0.001 and P < 0.05 respectively).

Sequencing of the voltage-gated sodium channel gene conducted on the Vauclin strain showed the presence of the *kdr *mutation at position 1016 (**G**TA to **A**TA) leading to the replacement of valine by an isoleucine (V1016Ile) at a high allelic frequency (f(R) = 0.71, n = 24) with RR = 12, RS = 11 and SS = 1. Conversely, no *kdr *resistant allele was detected in the susceptible Bora-Bora strain (n = 30).

**Figure 2:**
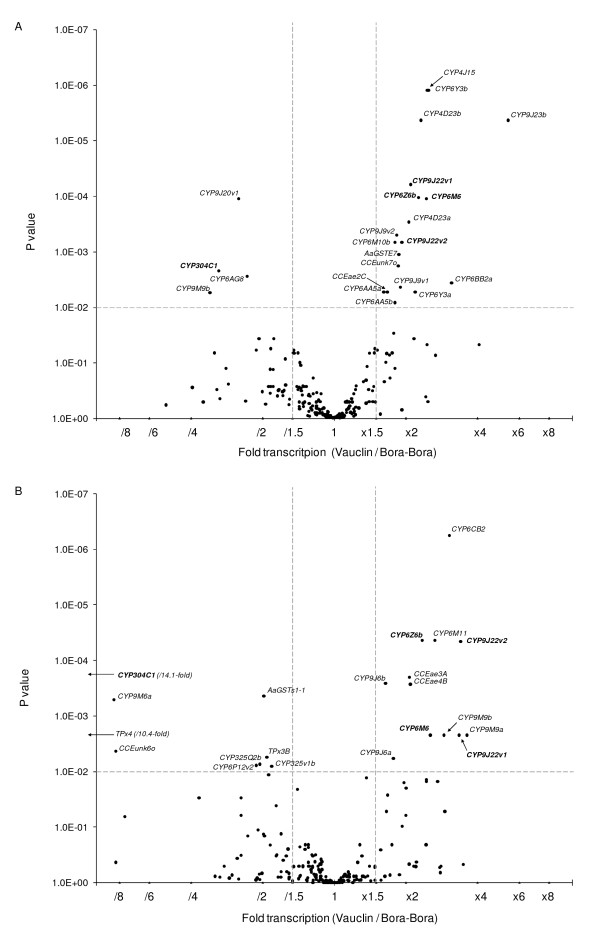
**Microarray screening of detoxifications genes differentially transcribed in the insecticide-resistant strain Vauclin**. Differential transcription of detoxification gens was investigated separately in 4^th^-stage larvae (A) and 3-days old adults (B). For each life stage, differences in gene transcription are indicated as a function of both transcription ratio (Vauclin/Bora-Bora) and ratio's significance (t-test P values). For each comparison, only probes showing consistent data in at least 3 hybridisations out of 6 were considered. Vertical lines indicate 1.5-fold transcription difference in either direction. Horizontal line indicates significance threshold (p < 0.01) adopted for the one sample t-test after Benjamini and Hochberg multiple testing correction procedure. Probes showing both more than 1.5-fold differential transcription and a significant P value are named. Probes that were found under- or over-transcribed in both larvae and adults are shown in bold. Suffixes *a *and *b *represent two different probes of the same gene while suffixes *v1 *and *v2 *represent two different alleles of the same gene.

We used the microarray '*Aedes Detox Chip*' (Strode et al., 2007) to compare the transcription levels of all *Ae. aegypti *detoxification genes between the insecticide-resistant strain Vauclin and the susceptible strain Bora-Bora in larvae and adults. Overall, 224 and 214 probes out of 318 were detected consistently in at least 3 hybridisations out of 6 in larvae and adults respectively. Among them, 31 detoxification genes were significantly differentially transcribed (transcription ratio > 1.5-fold in either direction and corrected P value < 0.01) in larvae or adults (Figure 2 and Additional file 1). Most of these genes encode P450s (*CYPs*) with 4 of them being differentially transcribed in the Vauclin strain at both life stages (*CYP9J22*, *CYP6Z6*, *CYP6M6 *and *CYP304C1*).

In larvae, 18 genes (15 *CYPs*, 1 *GST *and 2 *CCEs*) were found significantly differentially transcribed between the insecticide-resistant strain Vauclin and the susceptible strain Bora-Bora (Figure 2A). Among them, 14 genes were over-transcribed in the Vauclin strain while only 4 genes were under-transcribed. Most over-transcribed genes were represented by *CYP *genes with a majority belonging to the *CYP6 *subfamily (*CYP6BB2*, *CYP6M6*, *CYP6Y3*, *CYP6Z6*, *CYP6M10 *and *CYP6AA5*). Three *CYP9s *were also over-transcribed in larvae of the Vauclin strain (*CYP9J23*, *CYP9J22 *and *CYP9J9*) with a strong over-transcription of *CYP9J23 *(5.3-fold) together with 2 *CYP4s *(*CYP4J15 *and *CYP4D23*). Among other over-transcribed genes, 2 carboxy/cholinesterases (*CCEunk7o *and *CCEae2C*) and 1 glutathione *S*-transferase (*AaGSTE7*) were slightly over-transcribed in the Vauclin strain. Lastly, 4 *CYP*s (*CYP9M9*, *CYP9J20*, *CYP304C1 *and *CYP6AG8*) were under-transcribed in insecticide-resistant larvae comparatively to susceptible Larvae.

In adults, 18 genes (12 *CYPs*, 1 *GST*, 3 *CCEs *and 2 *Red/Ox*) were found differentially transcribed in the insecticide-resistant strain Vauclin comparatively to the susceptible strain Bora-Bora (Figure 2B). As in larvae, most of the over-transcribed genes belong to the *CYP6 *and *CYP9 *subfamilies (*CYP6CB2*, *CYP6M11*, *CYP6Z6*, *CYP6M6 *and *CYP9J22*, *CYP9M9*, *CYP9J6*) with only 2 additional *CCEs *(*CCEae3A *and *CCEae4B*) being moderately over-transcribed in the Vauclin strain. Nine genes were under-transcribed in Vauclin adults, including 5 *CYPs *(*CYP304C1*, *CYP9M6*, *CYP325Q2*, *CYP325V1 *and *CYP6P12*), 1 *CCE *(*CCEunk6o*), 1 *GST *(*GSTS1-1*) and 2 thioredoxin peroxidases (*TPx4 *and *TPx3B*). Interestingly *CYP304C1 *and *TPx4 *were both found strongly under-transcribed (14.1 and 10.4-fold respectively) in insecticide-resistant adults.

**Figure 3:**
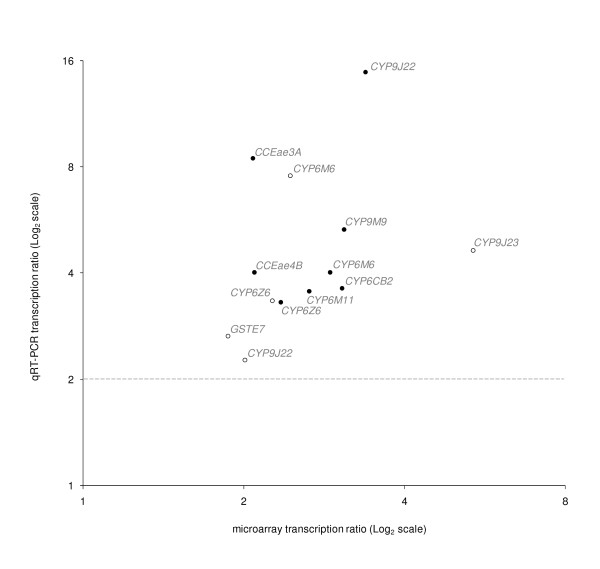
**Real-time quantitative RT-PCR validation of microarray data**. Validation of differential transcription between the two strains was performed on 11 selected genes in 4th-stage larvae (white dots) and 3-days old adults (black dots). Transcription ratios obtained from real-time quantitative RT-PCR experiments were normalized with the two housekeeping genes *AeRPL8 *and *AeRPS7 *and shown as mean value over 3 independent biological replicates.

Validation of microarray data was performed by real-time quantitative RT-PCR on 10 detoxification genes identified as over-transcribed in larvae or adults of the Vauclin strain (Figure 3). The over-transcription of genes identified from microarray experiments were all confirmed by quantitative RT-PCR in both life stages, although expression ratios obtained from RT-PCR were frequently higher than those obtained from microarray experiments.

## Discussion

The aim of the present study was to investigate insecticide resistance mechanisms of *Ae. aegypti *mosquitoes from Martinique (French West Indies).

Toxicological results confirmed the high level of resistance of the Vauclin strain from Martinique to the organophosphate temephos at the larval stage and to the pyrethroid deltamethrin at the adult stage [30]. The use of specific detoxification enzyme inhibitors suggested that resistance of larvae to temephos is linked to carboxylesterases and to a lesser extent P450s and GSTs. In adults, resistance to deltamethrin appeared principally linked to P450s and GSTs. Comparison of global detoxification enzyme activities between the two strains revealed elevated P450s, GSTs and in a lesser extent CCEs activities in the Vauclin strain at both life-stages, confirming the importance of metabolic resistance mechanisms in Martinique.

Carboxylesterases based-resistance mechanism is a major mechanism for organophosphate resistance in insects [12]. Several examples of *Ae. aegypti *resistance to organophosphates in the Caribbean linked to elevated carboxylesterases activities have been described [25,31]. Our toxicological and biochemical data confirms these observations despite a moderate elevated level of CCEs activities in the Vauclin strain. Among detoxification enzymes, P450s have been shown to play a major role in pyrethroid resistance in insects [8,10,32]. In Martinique, Marcombe *et al. *[30] suggested the involvement of P450s in the reduced efficacy of deltamethrin space-spray operations. Elevated GST levels have also been frequently associated with insect resistance to insecticides such as DDT and pyrethroids [33-35]. Our toxicological and biochemical data support the role of P450s and GSTs in insecticide resistance in Martinique.

At the molecular level, several mutations in the voltage-gated sodium channel gene have been associated with pyrethroid resistance in *Ae. aegypti *from Asian, Latin American and Caribbean countries [27,29,36]. Our results revealed a high frequency (71%) of the V1016I *kdr *mutation in *Ae. aegypti *populations from the community of Vauclin. The role of this mutation in pyrethroid resistance was clearly demonstrated by genotype-phenotype association studies [37]. The high frequency of the mutation, together with the incomplete effect of enzyme inhibitors in adults, supports a contribution of this *kdr *mutation in deltamethrin resistance.

Acetylcholinesterase (AChE) is critical for hydrolysis of acetylcholine at cholinergic nerve synapses and is a target for organophosphate and carbamate insecticides [38]. Altered AchE is an important resistance mechanism to organophosphates in many insects. Following the methods of Alout *et al. *[39] and Bourguet *et al. *[40], AChE activities of Vauclin mosquitoes were determined to investigate the presence of the G119S and/or F290V mutations. No insensitive AChE phenotypes were found in any of the mosquitoes tested (Corbel V., unpublished data), suggesting that organophosphate resistance of the Vauclin strain is rather due to detoxification enzymes unless other mutations occurred elsewhere in the Ace gene.

Our microarray screening identified 14 and 9 over-transcribed detoxification genes in larvae and adults of the Vauclin strain respectively. Among them, 4 P450s (*CYP6M6*, *CYP6Z6*, *CYP9J23 *and *CYP9J22*), the glutathione S-transferase *GSTe7 *and the carboxy/cholinesterase *CCEae3A *were all confirmed to be over-transcribed at both life-stages, supporting their involvement in insecticide-resistance. Other genes appeared more highly over-transcribed in adults (*CYP9J22*, *CYP9M9*, *CYP6M11*, *CCEae3A*) or in larvae (*CYP6M6*), suggesting that particular enzymes might be more specifically involved in resistance to one insecticide during a particular life-stage as argued by Paul *et al. *[41]. Validation of transcription profiles by real-time quantitative RT-PCR was successful for the 10 genes tested although expression ratios obtained with RT-PCR were often higher. The underestimation of transcription ratios obtained from microarray data is likely due to technical issues and has been previously evidenced in other studies [14,42].

Over-transcription of genes encoding P450s has been frequently associated with metabolic-based insecticide resistance mechanisms in insects [10]. In mosquitoes, the *CYP6Z *subfamily has been previously associated with response to pyrethroid, carbamates and organochlorine insecticides. In *Ae. aegypti*, *CYP6Z9 *has been found 4-fold over-transcribed in a permethrin-resistant strain collected in Northern Thailand [20]. In two recent studies, *CYP6Z8 *was also identified as inducible by permethrin and other pollutants [14,15]. In *An. gambiae*, *CYP6Zs *have been frequently found constitutively over-transcribed in permethrin- and DDT-resistant strains [19,21,43]. Recent studies demonstrated that the enzyme encoded by *An. gambiae CYP6Z1 *can metabolize the insecticides carbaryl and DDT while *CYP6Z2 *with a narrower active site, can only metabolize carbaryl [44,45]. Recently, another *An. gambiae *P450 (*CYP6P3*), was shown to be able to degrade pyrethroid insecticides [22]. The over-transcription of *CYP6Z6 *in the Vauclin strain may indicate the involvement of *Ae. aegypti CYP6Zs *in insecticide resistance in Martinique. However, the decisive demonstration of their capability to metabolize insecticides requires further investigations.

The association of *CYP6Ms *with metabolic resistance to pyrethroids has also been previously described in mosquitoes. In *Ae. aegypti *larvae, *CYP6M6 *and *CYP6M11 *were found inducible by permethrin and pollutants [14]. Although no *Aedes CYP6Ms *have been found constitutively over-transcribed in other insecticide-resistant strains, *An. gambiae CYP6M2 *was found significantly over-transcribed in various strains resistant to pyrethroids [21,46]. Recent studies indicate that *CYP6M2 *is able to metabolize pyrethroid insecticides (Stevenson B. personal communication). Our results suggest that *Ae. aegypti CYP6M6 *and *CYP6M11*, with protein sequences similar to *An. gambiae CYP6M2*, might also be involved in resistance of *Ae. aegypti *to pyrethroids in Martinique.

Finally, the glutathione S-transferase *GSTE7 *and the carboxy/cholinesterase *CCEae3A *were both found over-transcribed in both life-stages of the Vauclin strain. The role GSTs in resistance to chemical insecticides has been previously evidenced in insects with the enzyme encoded by *An. gambiae GSTE2 *metabolizing DDT [35,47,48] and the housefly *MdGST6-A *metabolizing two organophosphate insecticides [49]. In *Ae. aegypti*, GSTE2 also metabolises DDT and is over-transcribed in a pyrethroid and DDT-resistant strain from Thailand [35]. In 2008, Strode *et al. *[20] also revealed the over-transcription of *GSTE7 *in pyrethroid-resistant mosquitoes. Our results confirm that *GSTE7 *might have a role in insecticide resistance in *Ae. aegypti*. Over-production of carboxylesterases has been showed to play an important role in resistance to organophosphate insecticides in mosquitoes [50-53]. Elevated esterase activities conferring resistance to organophosphate insecticides has usually been linked to genomic amplification of specific alleles although gene over-transcription may also be involved [12]. Considering the high resistance of larvae of the Vauclin strain to temephos, over-transcribed *CCEs *represent good candidates for organophosphate metabolism in *Ae. aegypti*.

It has been suggested that insecticide resistance could be accentuated by the exposure of mosquito populations to pollutants and pesticides used in agriculture [14,15,54,55]. In Martinique, bananas, sugar cane, and pineapple represent important cultured surface areas often localized near mosquito breeding sites. These cultures have been submitted for decades to heavy use of insecticides such as the organochlorates aldrin, dieldrin and chlordecone and herbicides such as the triazine simazine, the pyridines paraquat and glyphosate [56]. This particular situation is likely to have contributed to the high resistance of *Ae. aegypti *to chemical insecticides and to the selection of particular detoxification genes in Martinique.

## Conclusion

We have identified multiple insecticide resistance mechanisms in *Ae. aegypti *mosquitoes from Martinique (French West Indies) significantly reducing the insecticidal activity of insecticides used for their control. Microarray screening identified multiple detoxification genes over-transcribed at both life-stages in resistant mosquitoes, suggesting their possible involvement in insecticide-resistance. Further experimental validation by using enzyme characterization and RNA interference will allow confirming the role of these genes in the resistance phenotype. As previously shown in mosquitoes [57], the epistasis between the *kdr *mutation and particular P450s genes is likely to contribute to the high level of resistance to pyrethroids in *Ae. aegypti *from Martinique and might seriously threatens the control of dengue vectors in the future. A better understanding of the genetic basis of insecticide resistance is an essential step to implement more effective vector control strategies in the field in order to minimize dengue outbreaks.

## Methods

### Mosquito strains

Two strains of *Ae. aegypti *were used in this study. The susceptible reference Bora-Bora strain, originating from Bora-Bora (French Polynesia) is free of any detectable insecticide resistance mechanism. An *Ae. aegypti *colony was established from wild field-caught mosquito larvae collected from individual houses in the community of Vauclin in Martinique (Vauclin strain). Larvae and adults obtained from the F1 progeny were used for bioassays, biochemical and molecular studies.

### Insecticides and detoxification enzyme inhibitors

Two technical grade compounds were used, representing organophosphate and pyrethroid classes of insecticides, temephos (97.3%; Pestanal™, Riedel-de-Haën, Seelze, Germany) and deltamethrin (100%; AgreEvo, Herts, United Kingdom). In addition, three classical detoxification enzyme inhibitors were used for larval and adult bioassays; piperonyl butoxide (PBO; 5-((2-(2-butoxyethoxy)ethoxy) methyl)-6-propyl-1,3-benzodioxole; 90% Fluka, Buchs, Switzerland) an inhibitor of mixed-function oxidases, tribufos (DEF; S,S,S-tributyl phosphorotrithioate; 98.1% Interchim, Montluçon, France) an inhibitor of carboxylesterases and in a lesser extent of glutathione S-transferases (GSTs) and chlorfenethol (DMC; 1,1-bis (4-chlorophenyl) ethanol; 98% Pestanal™, Riedel-de-Haën, Seelze, Germany) a specific inhibitor of GSTs.

### Larval bioassays

Larval bioassays were performed using a standard protocol described by the World Health Organization [58]. Bioassays were carried out using late third and early fourth-instar larvae of the Bora-Bora and Vauclin strains. For each bioassay, 20 larvae of each strain were transferred to cups containing 99 ml of distilled water. Five cups per concentration (100 larvae) and 5 to 8 concentrations of temephos diluted in ethanol leading to 0 to 100% mortality were used. For each concentration, 1 ml of temephos at the desired concentration was added to the cups. Control treatments of 1 ml of ethanol were performed for each test. Temperature was maintained at 27°C ± 2°C all over the duration of bioassays, and larval mortality was recorded 24 h after exposure. Three replicates with larvae from different rearing batches were made at different times and the results were pooled for analysis. Larvae were then exposed to the insecticide plus each enzyme inhibitor for 24 h. Dose of enzyme inhibitors were determined according to preliminary bioassays showing that the sub lethal concentrations of inhibitors were 1 mg/L, 1 mg/Land 0.008 mg/L for PBO, DMC and DEF respectively.

### Topical applications

The intrinsic activity of deltamethrin against adult mosquitoes was measured using forced contact tests to avoid any side effects linked to the insect behavior as recommended by the World Health Organization [59]. A volume of 0.1 μL of insecticide solution in acetone was dropped with a micro - capillary onto the upper part of the pronotum of each adult mosquito that was briefly anaesthetized with CO2 and maintained on a cold table. Doses were expressed in nanograms of active ingredient per mg of mosquito body weight. A total of 50 individuals (non blood fed females, 2 - 5 days old) were used per insecticide dose and for controls, with at least five doses leading to 0 to 100% mortality. Each test was replicated twice (n = 100 per dose) using different batches of insects and insecticide solutions. After treatment, mosquitoes were maintained at 27°C ± 2°C and 80% ± 10% relative humidity in plastic cups with honey solution provided. Mortality was recorded after 24 h. To assess the effect of detoxification enzyme inhibitors, each adult female was exposed to sub lethal doses of PBO (1000 ng/female), DEF (300 ng/female) and DMC (500 ng/female) 1 h prior to deltamethrin topical application following the same protocol described above.

### Mortality data analysis

Larval and adult mortality levels were corrected by the formula of Abbott [60] in case of control mortality > 5%, and data were analysed by the log-probit method of Finney [61] using the Probit software of Raymond *et al. *[62]. This software uses the iterative method of maximum likelihood to fit a regression between the log of insecticide concentration and the probit of mortality. The goodness of fit is estimated by a weighted χ^2^. It also estimates the slope of the regression lines and the lethal concentrations (LC_50 _and LC_95_for larvae) or dosages (LD_50 _and LD_95 _for adults) with their 95% confidence intervals. Bora-Bora and Vauclin strains were considered as having different susceptibility to a given pesticide when the ratio between their LC_50/95 _or LD_50/95 _(resistance ratio: RR_50/95_) had confidence limits excluding the value of 1. A mosquito strain is considered susceptible when its value of RR_50 _is less than 5, moderately resistant when RR_50 _is between 5 and 10, and highly resistant when RR_50 _is over 10. For detoxification enzyme inhibitors, synergism ratio's (SR_50 _and SR_95_) were obtained by calculating the ratio between the LC_50 _(or LD_50_) and LC_95 _(or LD_95_) of each insecticide with and without each enzyme inhibitor. A SR significantly higher than 1 indicated a significant effect of enzyme inhibitor and synergist effects were considered different between the two strains when their confidence interval (CI) were not overlapping.

### Detoxification enzyme activities

P450 monooxygenase activities were comparatively evaluated between susceptible and resistant strains in both larvae and adults by measuring the 7-ethoxycoumarin-O-deethylase (ECOD) activity on microsomal fractions based on the microfluorimetric method of De Sousa *et al*. [63]. One gram fresh 4^th ^stage larvae or 3 days-old adults (50% males and 50% females) were homogenised in 12 mL of 0.05 M phosphate buffer (pH 7.2) containing 5 mM DTT, 2 mM EDTA and 0.8 mM PMSF. The homogenate was centrifuged at 10000 g for 20 min at 4°C and the resulting supernatant was ultracentrifuged at 100000 g for 1 h at 4°C. The microsomal fraction was then resuspended in 0.05 M phosphate buffer and the microsomal protein content was determined by the Bradford method. Twenty μg microsomal proteins were added to 0.05 M phosphate buffer (pH = 7.2) containing 0.4 mM 7-ethoxycoumarin (7-Ec, Fluka) and 0.1 mM NADPH for a total reaction volume of 100 μL and incubated at 30°C. After 15 min, the reaction was stopped and the production of 7-hydroxycoumarin (7-OH) by P450 monooxygenases was evaluated by measuring the fluorescence of each well (380 nm excitation, 460 nm emission) with a Fluoroskan Ascent spectrofluorimeter (Labsystems, Helsinski, Finland) in comparison with a scale of 7-OH (Sigma). P450 activities were expressed as mean pmoles of 7-OH per mg of microsomal protein per min ± SE. Statistical comparison of P450 activities between the two strains was performed by using a Mann and Whitney test (N = 15).

Glutathione S-transferase activities were comparatively measured on 200 μg of cytosolic proteins from the 100000 g supernatant (see above) with 1-chloro-2,4-dinitrobenzene (CDNB, Sigma) as substrate [64]. Reaction mixture contained 2.5 mL of 0.1 M phosphate buffer, 1.5 μM reduced glutathione (Sigma), 1.5 μM CDNB and 200 μg proteins. The absorbance of the reaction was measured after 1 min at 340 nm with a UVIKON 930 spectrophotometer. Results were expressed as mean nmoles of conjugated CDNB per mg of protein per min ± SE. Statistical comparison of GST activities between the two strains was performed by using a Mann and Whitney test (N = 15).

Carboxylesterases activities were comparatively measured on 30 μg of cytosolic proteins from the 100000 g supernatant (see above) according to the method described by Van Asperen *et al*. [65] with α-naphthylacetate and β-naphthylacetate used as substrates (α-NA and β-NA, Sigma). Thirty μg cytosolic proteins were added to 0.025 mM phosphate buffer (pH 6.5) with 0.5 mM of α-NA or β-NA for a total volume reaction of 180 μL and incubated at 30°C. After 15 min, reaction was stopped by the addition of 20 μL 10 mM Fast Garnett (Sigma) and 0.1 M sodium dodecyl sulfate (SDS, Sigma). The production of α- or β-naphthol was measured at 550 nm with a Σ960 microplate reader (Metertech, Taipei, Taiwan) in comparison with a scale of α-naphthol or β-naphthol and expressed as mean μmoles of α- or β-naphthol per mg of cytosolic protein per min ± SE. Statistical comparison of esterase activities between the two strains was performed by using a Mann and Whitney test (N = 30).

### Kdr genotyping

Genomic DNA was extracted from whole adult mosquitoes of the Bora-Bora and Vauclin strains by grinding tissues with a sterile micro-pestle in DNA extraction buffer (0.1 M Tris HCl pH 8.0, 0.01 M EDTA, 1.4 M NaCl, 2% cetyltrimethyl ammonium bromide). The mixture was incubated at 65°C for five min. Total DNA was extracted with chloroform, precipitated in isopropanol, washed in 70% ethanol, and resuspended in sterile water. The *kdr *genomic region was amplified by PCR using Dip3 (5'-ATCATCTTCATCTTTGC-3') and Dip2A (5'-TTGTTGGTGTCGTTGTCGGCCGTCGG-3') primers. PCR steps included an initial denaturation step at 95°C for 3 min, followed by 45 cycles at 95°C for 30 s, 48°C for 30 s, and 72°C for 45 s, and a final extension step at 72°C for six min. PCR products were gel-purified with the QIAquick Gel Extraction Kit (Qiagen) before sequencing on an ABI Prism 3130 XL Genetic Analyser (Applied Biosystems) using the same primers.

### Microarray screening of differentially transcribed detoxification genes

The *Aedes detox chip *DNA-microarray, initially developed by Strode *et al. *[20] and recently updated with additional genes, was used to monitor changes in the transcription of detoxification genes between the Vauclin and the Bora-Bora strains in 4^th^-stage larvae and 3 days-old adults. This microarray contains 318 probes representing 290 detoxification genes including all cytochrome P450 monooxygenases (P450s), glutathione S-transferases (GSTs), carboxy/cholinesterases (CCEs) and additional enzymes potentially involved in response to oxidative stress from the mosquito *Ae. aegypti*. Each probe, plus 6 housekeeping genes and 23 artificial control genes (Universal Lucidea Scorecard, G.E. Health Care, Bucks, UK) were spotted 4 times at different positions on each array.

RNA extractions, cRNA synthesis and labeling reactions were performed independently for each biological replicate. Total RNA was extracted from batches of 30 4^th^-stage larvae or 30 3 days-old adults (15 males and 15 females) using the PicoPure™ RNA isolation kit (Molecular Devices, Sunnyvale, CA, USA) according to manufacturer's instructions. Genomic DNA was removed by digesting total RNA samples with DNase I by using the RNase-free DNase Set (Qiagen). Total RNA quantity and quality were assessed by spectrophotometry using a Nanodrop ND1000 (LabTech, France) and by using a Bioanalyzer (Agilent, Santa Clara, CA, USA). Messenger RNAs were amplified using the RiboAmp™ RNA amplification kit (Molecular Devices) according to manufacturer's instructions. Amplified RNAs were checked for quantity and quality by spectrophotometry and Bioanalyzer. For each hybridisation, 8 μg of amplified RNAs were reverse transcribed into labelled cDNA and hybridised to the array as previously described by David *et al. *[19]. For each life-stage, 3 pairwise comparisons of Vauclin strain versus Bora-Bora strain were performed with different biological samples. For each biological replicate, 2 hybridizations were performed in which the Cy3 and Cy5 labels were swapped between samples for a total of 6 hybridisations per comparison in each life-stage.

Spot finding, signal quantification and spot superimposition for both dye channels were performed using Genepix 5.1 software (Axon Instruments, Molecular Devices, Sunnyvale, CA, USA). For each data set, any spot satisfying one of the following conditions for any channel was removed from the analysis: (*i*) intensity values less than 300 or more than 65000, (*ii*) signal to noise ratio less than 3, (*iii*) less than 60% of pixel intensity superior to the median of the local background ± 2 SD. Data files were then loaded into Genespring 7.2 (Agilent Technologies, Santa Clara, CA USA) for normalization and statistic analysis. For each array, the spot replicates of each gene were merged and expressed as median ratios ± SD. Data from dye swap experiments were then reversed and ratios were log transformed. Ratio values below 0.01 were set to 0.01. Data were then normalized using the local intensity-dependent algorithm *Lowess *[66] with 20% of data used for smoothing. For each comparison, only genes detected in at least 50% of all hybridizations were used for further statistical analysis. Mean transcription ratios were then submitted to a one-sample Student's t-test against the baseline value of 1 (equal gene transcription in both samples). Genes showing a transcription ratio > 1.5-fold in either direction and a t-test P value lower than 0.01 after Benjamini and Hochberg multiple testing correction [67] were considered significantly differentially transcribed between the two strains.

### Real-time quantitative RT-PCR validation

Transcription profiles of 10 detoxification genes in 4^th^-stage larvae and adults were validated by reverse transcription followed by real-time quantitative RT-PCR on the same RNA samples used for microarray experiments. Four μg total RNAs were treated with DNAse I (Invitrogen) and used for cDNA synthesis with superscript III (Invitrogen) and oligo-dT_20 _primer for 60 min at 50°C according to manufacturer's instructions. Resulting cDNAs were diluted 125 times for PCR reactions. Real-time quantitative PCR reactions of 25 μL were performed in triplicate on an iQ5 system (BioRad) using iQ SYBR Green supermix (BioRad), 0.3 μM of each primer and 5 μL of diluted cDNAs according to manufacturer's instructions. For each gene analysed, a cDNA dilution scale from 5 to 50000 times was performed in order to assess efficiency of PCR. Data analysis was performed according to the ΔΔC_T _method taking into account PCR efficiency [68] and using the genes encoding the ribosomal protein L8 [GenBank DQ440262] and the ribosomal protein S7 [GenBank EAT38624.1] for a dual gene normalisation. For each life-stage, results were expressed as mean transcription ratios (± SE) between the insecticide-resistant strain Vauclin and the susceptible strain Bora-Bora. Only genes showing more than 2-fold over- or under-transcription in the Vauclin strain were considered significantly differentially expressed.

## Availability

Data Deposition:

The description of the microarray '*Aedes Detox Chip' *can be accessed at ArrayExpress  acc. No. A-MEXP-623.

All experimental microarray data can be accessed at 

## Authors' contributions

SM participated in toxicological and biochemical studies together with microarray screening and *kdr *genotyping and helped to draft the manuscript. RP participated in biochemical studies, microarray screening and RT-qPCR. FD participated in toxicological studies. SR participated in RT-qPCR and helped to draft the manuscript. JB participated in toxicological studies. CS participated in microarray study. CB participated in *kdr *genotyping and sequencing. AY coordinated field mosquito collection in Martinique and helped to draft the manuscript. HR helped to draft the manuscript and coordinated the microarrays studies. VC conceived of the study and participated in its design and coordination and helped to draft the manuscript. JPD participated in the design of the study and its coordination, performed microarray data analysis and conceived the manuscript. All authors read and approved the final manuscript.

## Supplementary Material

Additional file 1 **Microarray transcription data**. This table contains all transcription data obtained from microarray analysis between the insecticide-resistant strain Vauclin and the susceptible strain Bora-Bora. Transcription ratios (Vauclin/Bora-Bora) and their associated corrected t-test P values are indicated for each gene in 4^th^-stage larvae and 3-days old adults. Click here for file

## References

[B1] WHO (2006). Report of the Scientific Working Group on dengue.

[B2] Yaicharoen R, Kiatfuengfoo R, Chaeronviriyaphap T, Rongnoparut P (2005). Characterization of deltamethrin, resistance in field populations of *Aedes aegypti *in Thailand. J Vect Ecol.

[B3] Ponlawat A, Scott JG, Harrington LC (2005). Insecticide susceptibility of *Aedes aegypti *and *Aedes albopictus *across Thailand. J Med Entomol.

[B4] Jirakanjanakit N, Rongnoparut P, Saengtharatip S, Chareonviriyaphap T, Duchn S, Bellec C, Yoksan S (2007). Insecticide susceptible/resistance status in *Aedes *(Stegomyia) *aegypti *and *Aedes *(Stegomyia) *albopictus *(Diptera: Culicidae) in Thailand during 2003-2005. J Econ Entomol.

[B5] Jirakanjanakit N, Saengtharatip S, Rongnoparut P, Duchon S, Bellec C, Yoksan S (2007). Trend of Temephos resistance in *Aedes *(Stegomyia) mosquitoes in Thailand during 2003-2005. Environ Entomol.

[B6] Rodriguez MM, Bisset JA, De Armas Y, Ramos F (2005). Pyrethroid insecticide-resistant strain of *Aedes aegypti *from Cuba induced by deltamethrin selection. J Am Mosq Control Assoc.

[B7] Rawlins SC, Martinez R, Wiltshire S, Legall G (1998). A comparison of surveillance systems for the dengue vector *Aedes aegypti *in Port of Spain, Trinidad. J Am Mosq Control Assoc.

[B8] Hemingway J, Hawkes NJ, McCarroll L, Ranson H (2004). The molecular basis of insecticide resistance in mosquitoes. Insect Biochem Mol Biol.

[B9] Hemingway J, Field L, Vontas J (2002). An overview of insecticide resistance. Science.

[B10] Feyereisen R, Gilbert LI, Iatrou K, Gill S (2005). Insect cytochrome P450. Comprehensive Molecular Insect Science.

[B11] Ranson H, Hemingway J (2005). Mosquito glutathione transferases. Methods Enzymol.

[B12] Hemingway J, Karunaratne SH (1998). Mosquito carboxylesterases: a review of the molecular biology and biochemistry of a major insecticide resistance mechanism. Med Vet Entomol.

[B13] Despres L, David JP, Gallet C (2007). The evolutionary ecology of insect resistance to plant chemicals. Trends Ecol Evol.

[B14] Poupardin R, Reynaud S, Strode C, Ranson H, Vontas J, David JP (2008). Cross-induction of detoxification genes by environmental xenobiotics and insecticides in the mosquito *Aedes aegypti*: Impact on larval tolerance to chemical insecticides. InsectBiochem Mol Biol.

[B15] Riaz MA, Poupardin R, Reynaud S, Strode C, Ranson H, David JP (2009). Impact of glyphosate and benzo[a]pyrene on the tolerance of mosquito larvae to chemical insecticides. Role of detoxification genes in response to xenobiotics. Aquat Toxicol.

[B16] Djouaka RF, Bakare AA, Bankole HS, Doannio JMC, Coulibaly ON, Kossou H, Tamo M, Basene HI, Popoola OK, Akogbeto MC (2007). Does the spillage of petroleum products in *Anopheles *breeding sites have an impact on the pyrethroid resistance?. Malar J.

[B17] Holt RA, Subramanian GM, Halpern A, Sutton GG, Charlab R, Nusskern DR, Wincker P, Clark AG, Ribeiro JM, Wides R (2002). The genome sequence of the malaria mosquito *Anopheles gambiae*. Science.

[B18] Nene V, Wortman JR, Lawson D, Haas B, Kodira C, Tu ZJ, Loftus B, Xi ZY, Megy K, Grabherr M (2007). Genome sequence of *Aedes aegypti*, a major arbovirus vector. Science.

[B19] David JP, Strode C, Vontas J, Nikou D, Vaughan A, Pignatelli PM, Louis C, Hemingway J, Ranson H (2005). The *Anopheles gambiae *detoxification chip: A highly specific microarray to study metabolic-based insecticide resistance in malaria vectors. Proc Natl Acad Sci USA.

[B20] Strode C, Wondji CS, David JP, Hawkes NJ, Lumjuan N, Nelson DR, Drane DR, Karunaratne S, Hemingway J, Black WC (2008). Genomic analysis of detoxification genes in the mosquito *Aedes aegypti*. Insect Biochem Mol Biol.

[B21] Muller P, Donnelly MJ, Ranson H (2007). Transcription profiling of a recently colonised pyrethroid resistant *Anopheles gambiae *strain from Ghana. BMC Genomics.

[B22] Muller P, Warr E, Stevenson BJ, Pignatelli PM, Morgan JC, Steven A, Yawson AE, Mitchell SN, Ranson H, Hemingway J (2008). Field-caught permethrin-resistant *Anopheles gambiae *overexpress CYP6P3, a P450 that metabolises pyrethroids. Plos Genetics.

[B23] Vontas J, Blass C, Koutsos AC, David JP, Kafatos FC, Louis C, Hemingway J, Christophides GK, Ranson H (2005). Gene expression in insecticide resistant and susceptible *Anopheles gambiae *strains constitutively or after insecticide exposure. Insect Mol Biol.

[B24] Vontas J, David JP, Nikou D, Hemingway J, Christophides GK, Louis C, Ranson H (2007). Transcriptional analysis of insecticide resistance in *Anopheles stephens *i using cross-species microarray hybridization. Insect Mol Biol.

[B25] Rodriguez MM, Bisset J, De Fernandez DM, Lauzan L, Soca A (2001). Detection of insecticide resistance in *Aedes aegypti *(Diptera: Culicidae) from Cuba and Venezuela. J Med Entomol.

[B26] Rodriguez MM, Bisset J, Ruiz M, Soca A (2002). Cross-resistance to pyrethroid and organophosphorus insecticides induced by selection with temephos in *Aedes aegypti *(Diptera: Culicidae) from Cuba. J Med Entomol.

[B27] Brengues C, Hawkes NJ, Chandre F, McCarroll L, Duchon S, Guillet P, Manguin S, Morgan JC, Hemingway J (2003). Pyrethroid and DDT cross-resistance in *Aedes aegypti *is correlated with novel mutations in the voltage-gated sodium channel gene. Med Vet Entomol.

[B28] Macoris MD, Andrighetti MTM, Takaku L, Glasser CM, Garbeloto VC, Bracco JE (2003). Resistance of *Aedes aegypti *from the State of Sao Paulo, Brazil, to organophosphates insecticides. Memorias Do Instituto Oswaldo Cruz.

[B29] Saavedra-Rodriguez K, Urdaneta-Marquez L, Rajatileka S, Moulton M, Flores AE, Fernandez-Salas I, Bisset J, Rodriguez M, McCall PJ, Donnelly MJ (2007). A mutation in the voltage-gated sodium channel gene associated with pyrethroid resistance in Latin American *Aedes aegypti*. Insect Mol Biol.

[B30] Marcombe S, Carron A, Darriet F, Etienne M, Agnew P, Tolosa M, Yp-Tcha MM, Lagneau C, Yébakima A, Corbel V (2009). Reduced Efficacy of Pyrethroid Space Sprays for Dengue Control in an Area of Martinique with Pyrethroid Resistance. Am J Tropical Med Hyg.

[B31] Wirth MC, Georghiou GP (1999). Selection and characterization of temephos resistance in a population of *Aedes Aegypti *from Tortola. British Virgin Islands. J Am Mosq Control Assoc.

[B32] Brogdon WG, McAllister JC (1998). Insecticide resistance and vector control. Emerg Infectious Diseases.

[B33] Vontas JG, Small GJ, Hemingway J (2001). Glutathione S-transferases as antioxidant defence agents confer pyrethroid resistance in *Nilaparvata lugens*. Biochem J.

[B34] Enayati AA, Ranson H, Hemingway J (2005). Insect glutathione transferases and insecticide resistance. Insect Mol Biol.

[B35] Lumjuan N, McCarroll L, Prapanthadara LA, Hemingway J, Ranson H (2005). Elevated activity of an Epsilon class glutathione transferase confers DDT resistance in the dengue vector, *Aedes aegypti*. Insect Biochem Mol Biol.

[B36] Rajatileka S, Black WC, Saavedra-Rodriguez K, Trongtokit Y, Apiwathnasorn C, McCall PJ, Ranson H (2008). Development and application of a simple colorimetric assay reveals widespread distribution of sodium channel mutations in Thai populations of *Aedes aegypti*. Acta Trop.

[B37] Donnelly MJ, Corbel V, Weetman D, Wilding CS, Williamson MS, Black Wt (2009). Does kdr genotype predict insecticide-resistance phenotype in mosquitoes?. Trends Parasitol.

[B38] Anthony N, Rocheleau T, Mocelin G, Lee HJ, Ffrench-Constant R (1995). Cloning, sequencing and functional expression of an acetylcholineesterase gene from the yellow-fever mosquito *Aedes aegypti*. FEBS Letters.

[B39] Alout H, Berthomieu A, Hadjivassilis A, Weill M (2007). A new amino-acid substitution in acetylcholinesterase 1 confers insecticide resistance to *Culex pipiens *mosquitoes from Cyprus. Insect Biochem Mol Biol.

[B40] Bourguet D, Capela R, Raymond M (1996). An insensitive acetylcholinesterase in *Culex pipiens *(Diptera: Culicidae) from Portugal. J Econ Entomol.

[B41] Paul A, Harrington LC, Scott JG (2006). Evaluation of novel insecticides for control of dengue vector *Aedes aegypti *(Diptera: Culicidae). J Med Entomol.

[B42] Yuen T, Wurmbach E, Pfeffer RL, Ebersole BJ, Sealfon SC (2002). Accuracy and calibration of commercial oligonucleotide and custom cDNA microarrays. Nucleic Acids Res.

[B43] Nikou D, Ranson H, Hemingway J (2003). An adult-specific CYP6 P450 gene is overexpressed in a pyrethroid-resistant strain of the malaria vector *Anopheles gambiae*. Gene.

[B44] McLaughlin LA, Niazi U, Bibby J, David JP, Vontas J, Hemingway J, Ranson H, Sutcliffe MJ, Paine MJI (2008). Characterization of inhibitors and substrates of *Anopheles gambiae *CYP6Z2. Insect Mol Biol.

[B45] Chiu TL, Wen ZM, Rupasinghe SG, Schuler MA (2008). Comparative molecular modeling of *Anopheles gambiae *CYP6Z1, a mosquito P450 capable of metabolizing DDT. Proc Natl Acad Sci USA.

[B46] Müller P, Chouaibou M, Pignatelli P, Etang J, Walker ED, Donnelly MJ, Simard F, Ranson H (2008). Pyrethroid tolerance is associated with elevated expression of antioxidants and agricultural practice in *Anopheles arabiensis *sampled from an area of cotton fields in Northern Cameroon. Mol Ecol.

[B47] Ding Y, Hawkes N, Meredith J, Eggleston P, Hemingway J, Ranson H (2005). Characterization of the promoters of Epsilon glutathione transferases in the mosquito *Anopheles gambiae *and their response to oxidative stress. Biochem J.

[B48] Ortelli F, Rossiter LC, Vontas J, Ranson H, Hemingway J (2003). Heterologous expression of four glutathione transferase genes genetically linked to a major insecticide-resistance locus from the malaria vector *Anopheles gambiae*. Biochem J.

[B49] Wei SH, Clark AG, Syvanen M (2001). Identification and cloning of a key insecticide-metabolizing glutathione S-transferase (MdGST-6A) from a hyper insecticide-resistant strain of the housefly *Musca domestica*. Insect Biochem Mol Biol.

[B50] Vaughan A, Hemingway J (1995). Mosquito carboxylesterase Est alpha 2(1) (A2). Cloning and sequence of the full-length cDNA for a major insecticide resistance gene worldwide in the mosquito *Culex quinquefasciatus*. J Biol Chem.

[B51] Mourya DT, Hemingway J, Leake CJ (1993). Changes in enzymes titers with age in four geographical strains of *Aedes aegypti *and their association with insecticide resistance. Med Vet Entomol.

[B52] Bisset JA, Rodriguez MM, Hemingway J, Diaz C, Small GJ, Ortiz E (1991). Malathion and pyrethroid resistance in *Culex quinquefasciatus *from Cuba: efficacy of pirimiphos-methyl in the presence of at least three resistance mechanisms. Med Vet Entomol.

[B53] Raymond M, Chevillon C, Guillemaud T, Lenormand T, Pasteur N (1998). An overview of the evolution of overproduced esterases in the mosquito *Culex pipiens*. Royal-Society Discussion Meeting on Insecticide Resistance - from Mechanisms to Management: Apr 08-09 1998; London, England.

[B54] Boyer S, David JP, Rey D, Lemperiere G, Ravanel P (2006). Response of *Aedes aegypti *(Diptera: Culicidae) larvae to three xenobiotic exposures: larval tolerance and detoxifying enzyme activities. Environ Toxicol Chem.

[B55] Diabate A, Baldet T, Chandre F, Akoobeto M, Guiguemde TR, Darriet F, Brengues C, Guillet P, Hemingway J, Small GJ (2002). The role of agricultural use of insecticides in resistance to pyrethroids in *Anopheles gambiae *s.l. in Burkina Faso. Am J Trop Med Hyg.

[B56] Bocquene G, Franco A (2005). Pesticide contamination of the coastline of Martinique. Marine Poll Bull.

[B57] Hardstone MC, Leichter CA, Scott JG (2009). Multiplicative interaction between the two major mechanisms of permethrin resistance, kdr and cytochrome P450-monooxygenase detoxification, in mosquitoes. J Evol Biol.

[B58] WHO (2005). Guidelines for laboratory and field testing of mosquito larvicides.

[B59] WHO (2006). Guidelines for testing mosquito adulticides for indoor residual spraying and treatment of mosquito nets.

[B60] Abbott W (1925). A method of computing the effectiveness of an insecticide. J Econ Entomol.

[B61] Finney DJ (1971). Probit analysis.

[B62] Raymond M (1993). PROBIT software. CNRS UMII, Licence L93019 Avenix, France.

[B63] De Sousa G, Cuany A, Brun A, Amichot M, Rhamani R, Bergé JB (1995). A microfluorimetric method for measuring ethoxycoumarin-*O*-deethylase activity on individuals *Drosophila melanogaster *abdomens: Interest for screening resistance in insect populations. An Biochem.

[B64] Habig H, Pabst MJ, Jacoby WB (1974). Gluthatione *S*-transferases: The first step in mercapturic acid formation. J Biol Chem.

[B65] Van Asperen K (1962). A study of housefly esterases by means of sensitive colorimetric methode. J Insect Physiol.

[B66] Cleveland WS, Devlin SJ (1988). Locally Weighted Regression - An approach to regression-Analysis by local fitting. J Am Stat Assoc.

[B67] Benjamini Y, Hochberg Y (1995). Controlling the False Discovery Rate: a Practical and Powerful Approach to Multiple Testing. J Royal Stat Soc B.

[B68] Pfaffl MW (2001). A new mathematical model for relative quantification in real-time RT-PCR. Nucl Acids Res.

